# Supplementary material for: Access to essential medicines for noncommunicable diseases during conflicts: The case cardiovascular diseases, diabetes and epilepsy in Northern Syria
Source: PLOS Glob Public Health. 2025 Dec 4;5(12):e0004744. doi: 10.1371/journal.pgph.0004744 (PMC12677547; doi:10.1371/journal.pgph.0004744)
Supplement: S1 Appendix — (DOCX) [file pgph.0004744.s001.docx]

**S1 Appendix: Interviews topic guides with study participants**

**Topic guide for interviews with patients**

| **Domain** | **Topic and Probes** |
| --- | --- |
| Background information | - Age and sex of the participant - Which NCDs they have? - When the these NCDs were diagnosed? |
| Used medicines | - Which medicines are they using? - Sources of medicines (Prescribed, without prescription, shared…) |
| Perception of availability | - Where do they get the medicines from? (Pharmacy, shared, other healthcare facilities, other) - Were these medicines always available in accessible pharmacies or other facilities? - In case of stockouts or shortages of these medicines, what did they do? Who did they ask for help? - What are reasons behind stockouts or shortages of medicines? - How did the conflict affect the availability of these medicines? - Did the economic sanctions affect the availability of medicines? If yes, how? - How did the recent earthquake and its consequences effected the availability of medicines? |
| For insulin-dependent diabetic patients | - Access to syringes and glucometers. |
| Perception of price | - Do you pay out of pocket for the medicines you need? - Considering the medicines you use, how did the conflict affect the prices of these medicines? - How do you navigate the system to find outlets where you could get medicines with “better or fair price”? - Do you have an understanding of the rationale behind the pricing of the medicines you need? If yes, can you explain? - Did the prices of medicines change after the recent earthquake? How? - Did the sanctions imposed on Syria impact the prices of medicines? |
| Perception of Quality | - Are you satisfied with the quality of the medicines you use? - Have you ever experienced using a medicine with a “bad quality”? How did you decide that this medicine was of “bad quality”? Did you report this to anybody? - How do you decide on the quality of medicine? - Which resources of medicines you trust/do not trust when it comes to assuring the quality of the medicines you need? - How did the quality of the medicines you use influenced by different events (Conflict, economic sanctions, earthquake? |
| Adherence and challenges to adherence | - Looking over the past three months, did you adhere to the medicines you need? Or there were some interruptions? - What were the reasons for none-adherence? |
| Learned lessons | - What are the lessons you have learned from the previous challenges? |
| Other comments | - Do you have any additional comments? |

**Topic guide for interviews with pharmacists**

| **Domain** | **Topic and Probes** |
| --- | --- |
| Background information | - Age and sex of the participant - Qualification - Type of the facility the pharmacist work in. - Located in rural or Urban area? |
| Organization of pharmaceutical System | - Which “local authorities” are responsible for organizing the pharmaceutical sector? - Sources of medicines and medicines distributors in the region. - What - How is the practice of pharmacists regulated in your region? Which authorities are responsible for regulating this? |
| Role of Pharmacists in primary care during conflict | - How did the role of pharmacists changed over the different shocks (conflict, sanctions, earthquake) in providing access to primary care? |
| Perception on availability of medicines | - Which medicines are commonly in short supply? - What are the reasons behind medicines shortages? - What are the strategies you follow to avoid medicines shortages? Any criteria for prioritization? |
| Perception on Price | - How are the prices of medicines are regulated? - How did the different shocks (conflict, sanctions, earthquake) affect medicines prices? |
| Perception of Quality | - Who is responsible for medicines quality assurance in the region you work in? - How do you assess/assure the quality of medicines you procure for your facility? - Do patients complain about medicines quality? - Are you aware of certain medicines that are more likely to be substandard or falsified (SF)? - Do you report any SF medicines to any authority? Which one? - Who is responsible for withdrawing SF medicines from the market? |
| Learned lessons | - The lessons you have learnt through your experiences during various challenges or shocks to maintain or improve access to medicines for your community. |
| Other comments | - Do you have any additional comments? |

**Topic guide for interviews with other stakeholders**

| **Domain** | **Topic and Probes** |
| --- | --- |
| Background information | - Age and sex of the participant - Qualification - Role in the health System |
| Role in health system | - What is the role you/your organization play in providing access to medicines? - How did the role of your organization or facility changed over the different shocks (conflict, sanctions, earthquake) in providing access to medicines? - Collaboration between your organization or facility and facilities to provide access to medicines. |
| Perception on availability of medicines | - How is the medicines supply chain organized and maintained in the region you work in? - Role of your facility in maintaining availability of medicines. |
| Perception on Price | - How are the medicines prices regulated in the region you work in? - Role of your facility in maintaining affordability of medicines. |
| Perception of Quality | - Who is responsible for medicines quality assurance in the region you work in? - Role of your organization in medicines quality assurance. - Who is responsible for withdrawing SF medicines from the market? |
| Learned lessons | - The lessons you have learnt through your experiences during various challenges or shocks to maintain or improve access to medicines for your community. |
| Other comments | - Do you have any additional comments? |
