# Supplementary material for: Access to essential medicines for noncommunicable diseases during conflicts: The case cardiovascular diseases, diabetes and epilepsy in Northern Syria
Source: PLOS Glob Public Health. 2025 Dec 4;5(12):e0004744. doi: 10.1371/journal.pgph.0004744 (PMC12677547; doi:10.1371/journal.pgph.0004744)
Supplement: Attachment — Submitted filename: Response to Reviewers.docx [file pgph.0004744.s003.docx]

5. Review Comments to the Author

**Reviewer #1: Thank you for the opportunity to review your well conducted, well written study. I commend you for shedding the light on a global health topic of extreme importance and using a current, prolonged, ongoing conflict to provide valuable insights on reality of access to medicine in conflict zones.**

Thank you so much! We appreciate your encouraging comments on our study and thank you for your helpful feedback. We believe that our manuscript is stronger after considering your helpful suggestions and comments.

**The abstract clearly communicates the study's objectives, methods, key findings, and implications.
The background is rich, well-informed, and cites a substantial body of research. It effectively situates the study moving from a global perspective to a specific national context. The authors offer a concise description of the contemporary healthcare reality in Syria and how it has drastically changed because of the recent civil war. The Syrian context is presented as a case study that highlights challenges faced in conflict zones at large and thus offer valuable insights for concerned policy makers.

The authors chose to focus on three NCD: HTN, DM and Epilepsy. The choice is justified in the methods section
The study adapts WHO/HAI methodologies with explained deviations to match the context of Northern Syria, accounting for specific limitations in healthcare infrastructure, regulatory frameworks, and licensing. The integration of quantitative and qualitative phases each with distinct protocols and rationale, strengthens the study's credibility and offers additional contextual depth to interpret the results.
The section addresses the ethical challenges of working in a conflict zone in a transparent and thorough way.**

We thank the reviewer for the encouraging and detailed feedback on various aspects of the study, which we greatly appreciate.

**A potential for bias in data sampling should be acknowledged:
- Participants were guided by the investigators during the quantitative data collection (line 179). The supplement file lists the targeted questions. Clarify if one or more of the investigators were involved in these virtual sessions, and if the responders training was standardized. This step might introduce a potential for bias in the reported answers.**

Thank you for raising this point. The principal investigator (SA) conducted the quantitative data collection. All respondents received standardized instructions and background information regarding the data collection process. The virtual sessions with the principal investigator aimed to clarify any uncertainties, support respondents during completion, and minimize data entry errors. We agree that this guidance may have introduces a potential response bias. While our intention was to enhance data quality and consistency, we acknowledge this limitation and have added the following statement in the manuscript to reflect on this limitation:

“*Additionally, the involvement of the principal investigator (SA) in guiding respondents through virtual sessions may have introduced a potential for response bias, as respondents could have been unintentionally influenced by the investigator’s (digital) presence or perceived expectations. While this approach aimed to ensure consistency and clarity in a challenging remote setting, it may have influenced how participants interpreted or responded to some items*.” (Lines 689-693 of the track-changes version)

**- The used grounded theory methodology adds credibility to the authors' data. It is noted that two investigators transcribed the interviews, but only translated the identified codes for the other research team members (line 223). This step might introduce a potential bias in data analysis.**

Thank you for this thoughtful observation. To facilitate collaborative analysis within the wider team, the identified codes and emerging themes were translated into English. This approach balanced contextual depth with inclusive team engagement, given the background and linguistic diversity in the team. However, we acknowledge that translating only selected codes may have limited the co-authors’ access to the full richness of participants’ narratives and introduced a potential for misinterpretation. We have added the following to reflect on this potential limitation:

“*Only the identified codes were translated into English to facilitate collaborative analysis among co-authors, given the background and linguistic diversity in the research team. While this approach supported inclusive interpretation, it may have limited the wider team’s access to the full contextual richness of participants’ original narratives, introducing a potential risk of misinterpretation or analytic bias*.” (Lines 694--698 of the track-changes version)

**- Snowball sampling method was probably necessary given the conditions on the ground in Northern Syria. However, a potential for sampling bias should be acknowledged.**

To address this point, we have added the following sentence to the limitations section of the manuscript:

“*While the use of snowball sampling may introduce some risk of sampling bias, the inclusion of a wide range of public and private health facilities across urban and rural areas in four regions of Northern Syria likely mitigates this limitation to some extent*.” (Lines 698-700 of the track-changes version)

**- Consider adding a statement if any special requirements were issued by the Tropical Institute IRB given the fragile/conflict settings of the sampled population.**
Thank you for your suggestion. To address your suggestion, we added the following to the ethics section:

“*Additionally, as part of their review of the protocol, the IRB specifically asked the research team to identify psychological support services (e.g., NGO-operated programs) to which participants who experienced any form of distress could be referred, recognizing the limitations of such services in Syria. This measure was implemented, and where formal support was unavailable due to the local circumstances, the use of culturally appropriate self-help materials, such as those developed by the WHO, was applied as an alternative*.” (Lines 285-290 of the track-changes version)

**Results:
The qualitative results are clearly reported. The analysis covers several crucial and interrelated themes, which are organized in subsections in a logical manner. The themes are grounded in data with several supporting (translated) quotes from the participants.

This discussion section is rich and structured. It situates the study results into the broader topic of medicine access in a conflict-affected setting. Quantitative and qualitative results are effectively synthesized, with comparison to other conflict zones. The impact of systemic drivers such as geopolitical instability, and regulatory collapse on pharmaceutical access is aptly discussed. A call to action for further research into healthcare in conflict-zones highlights the added value of this study and offers suggestions for others to build on the author's experience.**
We thank the reviewer for the encouraging and detailed feedback on our results and discussion sections.

**Strength and Limitations are honestly discussed. I invite the authors to reflect on the additional limitations mentioned above in the methodology section.**
As advised, and as described in our answers above, we have added several points to the limitations section to reflect the methodological considerations raised.

**A clear summary is provided in the conclusion section and emphasis on practical recommendations for policy makers and future research.

Other comments:
Line 79: health workers "fleeing" the country**

Thank you for pointing this out. We have replaced “fleeing” with “fled” (Line 70 of the track-changes version)

**Line 197: “international price reference guidelines has not been updated” → “have not been updated”**
We have corrected the verb agreement as suggested.

**I commend the authors on their hard work.**

We sincerely thank the reviewer for their encouraging words and thoughtful review.

**Reviewer #2: Thank you for the opportunity to provide feedback to this important work.**
We thank the reviewer for their time and engagement with our work.

**Introduction: Lines 124-127. I imagine another primary goal of the research is to conduct a gap analysis for the Syrian health care system to allocate resources and better meet the treatment needs of the Syrian population.**

We thank the reviewer for this thoughtful suggestion. While we agree that the findings may help inform gap analyses and resource allocation decisions, we have opted not to add this as a primary aim at the reporting stage, as this would not be consistent with the study’s main aims as defined in the IRB-approved protocol. That said, we recognize the relevance of this perspective. We note that the ITM Pharmaceutical Public Health Unit has a specific research line on access to medicines in Syria (and other conflict settings) and we look forward to developing these aspects in future research.

**Methods- Lines 163-165. Authors omitted epilepsy medication, which the clearly analyzed.**

We thank the reviewer for this helpful observation. We have now explained the selection strategy for epilepsy medicines in the methods section as following:

“*The selection of medicines for cardiovascular diseases and diabetes was based on three complementary strategies: prior evidence of common prescription patterns [11], inclusion in the tenth Syrian National Essential Medicines List, and input from the advisory board (as described below) and HAI expert. For epilepsy essential medicines, the selection was guided by the tenth Syrian National Essential Medicines List, by the advisory board and by the HAI expert inputs, as no data on medicine use or prescribing patterns for epilepsy in Syria were available*.” (Lines 170-178 of the track-changes version)

**Despite the explanation of the essential medicines, it’s unclear (and seems a little random) why the authors chose cardiovascular and anti-epilepsy drugs.**

We thank the reviewer for this comment. However, we respectfully note that the rationale for focusing on cardiovascular diseases, diabetes, and epilepsy is explained in the introduction of our manuscript. Our introduction states that the selection was guided by three interrelated considerations: (1) their epidemiological burden in Syria, where NCDs account for a growing share of morbidity and mortality (Lines 55-69 of the track-changes version); (2) their historical neglect in humanitarian response frameworks, which have largely prioritized acute and infectious diseases (Lines 110–115 of the track-changes version); and(3) the heightened vulnerability of patients with these conditions in conflict settings due to the need for continuous treatment and the risk of complications when medicines are unavailable (Lines 107-113 of the track-changes version).

**The WHO 2023 list of essential medicine include: Non-steroidal anti-inflammatory medicine, psychotropic medicine, anti-allergic medicine, antidotes for poison, antibiotics/anti-viral/anti-fungal, anti-Parksinson, chemotherapy/immunodulators, contraceptives, vitamins/supplements, etc… It’s unclear why these were not examined.**

The WHO 23rd Model List of Essential Medicines includes over 500 medicines. Including the full list in a medicines availability and price survey would not be feasible. In fact, the widely-used standardized [WHO/HAI methodology](https://haiweb.org/what-we-do/price-availability-affordability/collecting-evidence-on-medicine-prices-availability/) for measuring medicine prices, availability, and affordability explicitly recommends a more focused approach, stating: “*To make the survey manageable, up to 50 medicines will be surveyed*.”

Notably, the WHO/HAI methodology refers to stable contexts. In our study, focusing on an unstable context, we selected 28 essential medicines to balance the need for breadth within the three NCDs of interest (cardiovascular diseases, diabetes, and epilepsy) and the operational feasibility of the survey, especially given the challenges of working in conflict-affected areas. The rationale for focusing on these three NCDs is detailed in the manuscript and elaborated further in our response to the previous comment.

**Lines 225-227. It’s unclear who conducted the codes and analysis of the qualitative data.**

The initial thematic analysis of the Arabic transcripts was conducted by SA and SF. Codes were developed based on this analysis, and translated into English, which enabled all co-authors, to review the codes, categories, and emerging themes. This process is now elaborated in the revised methods section as following:

“*SA and SF conducted the initial thematic analysis of the Arabic transcripts including development of a set of pertinent codes. The identified codes were then translated into English to enable all co-authors, including those not involved in data collection, to actively engage in the analytic process. Co-authors reviewed and provided feedback on the defined codes, categories, and emerging themes. Final themes were refined through iterative feedback until consensus was reached*.” (Lines 238-244 of the track-changes version)

**In line 225 it seems like the other co-authors conducted some of the coding. However, again, the PI’s involvement in the interviewing and coding does bias interpretation. How did multiple coders converge on themes? Were there consensus meetings? An adjudication process?**

We appreciate the reviewer’s concern about potential bias due to the PI’s dual role. While the PI (SA) and a co-author (SF) were involved in both data collection and initial coding, we sought to mitigate bias through collaborative analysis. As explained in the previous response, co-authors who were not involved in data collection reviewed and provided comments on the codes, categories and themes. Through iterative feedback across the team, consensus on final categories and themes was reached and analytical decisions were shaped collectively.

**Was any specialized software (NVivo or Atlas) used or were they collected in Excel?**

No specialized qualitative software was used. All codes, categories, and emerging themes were documented and organized using Microsoft Excel, which was deemed appropriate given the size of the dataset and the collaborative nature of the analysis. We have added this clarification to the methods section.

“*The coding and thematic analysis were conducted using Microsoft Excel.”* (Lines 244-245 of the track-changes version)

**Was a semi-structured questionnaire developed? If so, then the questionnaire should be included in the supplemental tables.**

Yes, a semi-structured questionnaire was developed and used for the qualitative interviews. This tool has already been included in the supplementary materials as S1 Appendix. A reference to this supplementary material was included in the methods section of the manuscript initially submitted (Line 228 of the track-changes version).

**In comparing northeast vs northwest and private vs public %, the authors could have conducted a simple pair wise chi squared analysis.**

We thank the reviewer for this suggestion. In line with the gold standard WHO/HAI standard methodology, we did not conduct inferential statistical analyses (e.g. chi-squared tests) for grouped comparisons across sectors or regions. Given the exploratory nature of our study in a complex conflict setting, we chose to prioritize descriptive analyses and contextual interpretation, to ensure methodological robustness and adherence to internationally accepted standards in this specific field of investigation.

**- Results: Lines 304-314 and Table 2. No units are provided is it USD per 100 units, per tablet, per prescription.**

Thank you for this observation. In our manuscript, we described each ‘medicine unit’ as a package (e.g., Captopril 25 mg (20 tablets)), meaning that prices referred to the unit = package, rather than the unit = tablet. However, we recognize that this may not have been sufficiently explicit. To correct this, we have revised the text to clearly indicate that the prices refer to the units = packages. For example, we changed the sentence on lines 331-332 of the track-changes version to read:

*“For example, a dispensing unit (package) of Captopril 25 mg (20 tablets) was priced at 1.39 USD [1.07–1.70] in Northwest Syria.”*

Additionally, we revised the title of Table 2 to clarify this further. The new title reads:

“*Median price [the 25th–75th Percentile] of lowest priced generic essential medicines packages (private pharmacies, n = 20).*” (Lines 240-241 of the track-changes version)

We also added the following to the methods section to clarify this point:

“*Availability and prices were recorded per package, and the package was used as the unit of analysis for both availability and price assessments*” (Lines 140-141 of the track-changes version)

**In the discussion, this needs to be contextualized to how the average Syrian can or can not afford. How much is the average salary and expenses of a Syrian? The cost by USD is not very meaningful with out context.**

We agree that contextualizing medicine prices in relation to income and living costs is essential for understanding affordability. In response, we have added the following statement to the discussion:

“*As a general reference point, the average monthly salary in Syria was reported to be around 81 USD in December 2020 , highlighting the severe economic hardship facing households over the past years*” (Lines 596-598 of the track-changes version)

As mentioned in the manuscript, this study is part of a broader project that included qualitative interviews with NCD patients. These interviews explored patient experiences with accessing and affording medicines in Northern Syria. As stated in the manuscript:

“*this project included qualitative interviews with NCD patients that explored their experiences with medicine affordability. Perceived affordability among patients will be analyzed and reported in a separate manuscript, providing further insights into the financial barriers to accessing medicines in this conflict-affected setting*.” (Lines 598-601 of the track-changes version)

**Discussion:
- Lines 485-487 the authors only cite two studies from Yemen and Afghanistan without any detail. Please compare the data. Also, many more developing countries that are not in conflict have limited supplies of essential medications. It’s worth comparing Syria to those numbers.**

Thank you for this important observation. In the original manuscript, we cited studies from Yemen and Afghanistan primarily to highlight that availability of essential medicines can be significantly affected by conflict-related factors, as has been observed in other fragile settings.

In response to your suggestion, we have now added to the discussion the following comparison of the availability of selected medicines between our study in Syria and the studies from Yemen and Afghanistan:

“*For instance, the average availability of furosemide was 70% in Syria, 70% in Yemen, and 61% in Afghanistan, while the availability of atorvastatin was 48% in Syria, compared to 57% in Yemen and 39% in Afghanistan*.” (Lines 513-515 of the track-changes version)

However, we urge caution in interpreting these comparisons due to important contextual and methodological differences across the studies. Specifically:

- Differences in surveyed facilities: The surveys were conducted in different types of health facilities.

- Differences in medicine lists: The selection of medicines varied substantially.

- Differences in dosage forms and strengths: The specific formulations and dosages surveyed differed across studies, which further limits direct comparability.

We have included this brief discussion of these comparisons in the manuscript while clearly outlining these limitations:

“*However, these comparisons should be interpreted with caution due to several key differences. First, the data were collected from different types of facilities: our study surveyed public hospital pharmacies, primary healthcare centers run by humanitarian organizations, and private pharmacies, whereas the study from Yemen included government hospitals, PHCs, and private pharmacies, and the study from Afghanistan included public and private pharmacies. Second, the selection of medicines varied across studies, with different emphasis on NCDs. Third, the formulations and dosages of medicines were not standardized across the three studies. Despite these limitations, the broadly similar patterns underscore the systemic impact of conflict on the availability of essential medicines*.” (Lines 515-523 of the track-changes version)

**- Lines 643- I think that it’s a bit much to label this study as “comprehensive and nuanced”. Perhaps the mixed methods offered a “holistic” appreciation of the challenges Syrians face in accessing essential medicine.**

We have revised the sentence to better reflect the contribution of our study with its mixed-methods approach and have replaced it with the following:

“*By combining both qualitative and quantitative methods, our mixed-methods approach provided a holistic perspective on the barriers to accessing essential medicines in the country*” (Lines 678-680 of the track-changes version)

**- Limitations include that the PI of the study conducted the interview. Most of the time interviews are not conducted by the PI to avoid bias on how the data is collected.**

We have added the following to report and reflect on this limitation:

“*Additionally, the involvement of the principal investigator (SA) in guiding respondents through virtual sessions may have introduced a potential for response bias, as respondents could have been unintentionally influenced by the investigator’s (digital) presence or perceived expectations. While this approach aimed to ensure consistency and clarity in a challenging remote setting, it may have influenced how participants interpreted or responded to some items*.” (Lines 689-693 of the track-changes version)

**- Minor feedback: Please check grammar throughout. For instance “low medicines”. Medicine can be itself a plural; throughout the text the authors use medicines, sometimes not appropriately. Language could be a bit tighter. For instance, “Our qualitive data suggested…” could be “Qualitative reports suggest…”**

Thank you for your careful reading and helpful suggestions. We reviewed the manuscript thoroughly and have made several corrections to improve grammar, and ensure appropriate use of "medicine" vs. "medicines." Specifically, we corrected instances where “medicines” was inappropriately used in place of the uncountable noun “medicine,” including lines 26, 74, 370, 396,398, 510,529, 537, 605, 629, and 704 of the track-changes version. We also revised phrasing for clarity, such as changing “*Our qualitative data suggested*…” to “*Qualitative reports suggested*…” (Line 30 of the track-changes version).

**- Figures/Tables: Line 84-96: consider adding a map to help the reader better understand the differential regional impact.**

Thank you for this helpful suggestion. We have added a map to illustrate the territorial control across Syria. The map appears as Figure 1 in the relevant section.

**- Table 1. It’s doesn’t make sense to take the decimal to the hundredth. Keep it to the tenth. It’s kind of a busy table. Consider color coding cells by low availability (30%–49%), and very low availability (<30%); maybe a pink and darker ping (red maybe too dark).**

Thank you for these suggestions. We have updated Table 1 (and throughout the manuscript) to display values rounded to one decimal place (tenth) instead of two (hundredth). In addition, we implemented color coding as recommended.

**- Table 2. Need to label that this is in USD.**

Thank you for pointing this out. We have clearly now indicated that all values in table 2 are presented in USD. (Line 341 of the track-changes version)
