# Supplementary material for: Access to essential medicines for noncommunicable diseases during conflicts: The case cardiovascular diseases, diabetes and epilepsy in Northern Syria
Source: PLOS Glob Public Health. 2025 Dec 4;5(12):e0004744. doi: 10.1371/journal.pgph.0004744 (PMC12677547; doi:10.1371/journal.pgph.0004744)
Supplement: Attachment — Submitted filename: Response_to_Reviewers_auresp_2.docx [file pgph.0004744.s004.docx]

We sincerely thank the reviewers and editors for their insightful comments, which have greatly helped us improve the overall quality of the manuscript. As explained below, we have carefully addressed each comment point by point and revised the text accordingly.

Reviewer #2: Methods-
**Selection of essential medicine (Lines 167-178) still not adequately answered. The revision gave very vague answer "prior evidence of common prescription patterns, inclusion in the tenth Syraina National Essential medicine list, and input from advisory board". As I previously commented, the selection of cardiovascular, diabetic, and epilepsy meds seems random. The authors did not adequately address this. At this point, they should at least acknowledge in the limitation section that perhaps due to access, availability of information, time and resources, other essential medicine (such as those listed) could not be evaluated.**

Thank you for this follow-up comment. We added the following to the methodology section to acknowledge the operational constraints that shaped our focus on essential medicines for cardiovascular, diabetes, and epilepsy:

“*The selection of surveyed essential medicines was necessarily restricted to cardiovascular, diabetes, and epilepsy medicines due to practical considerations related to feasibility of conducting the survey in conflict-affected areas. Consequently, other essential medicines for conditions outside these groups, although very important, could not be evaluated within the scope of this study*.” (Lines: 697-701 in the manuscript with track changes)

Discussion-
**Regarding my feedback "Lines 485-487 the authors only cite two studies from Yemen and Afghanistan without any detail. Please compare the data. Also, many more developing countries that are not in conflict have limited supplies of essential medications. It’s worth comparing Syria to those Numbers" my point was that the discussion was restricted to comparing to Yemen and Afghanistan. However, most developing countries also have challenges to access to essential medications. Please do not restrict the discussion to these two countries. Expand the discussion by comparing to other countries' access to essential medications.**

Thank you for this suggestion. In response, we expanded the discussion section to include additional comparisons with both another conflict-affected region (Gaza) and several low- and middle-income countries (LMICs) (Tanzania, Ethiopia, and Kenya). The revised text now reads:

*“Beyond conflict-affected settings, evidence from LMICs also reveals considerable variability in the availability of essential medicines for NCDs, with some medicines reported to have high availability while others were reported to have low or very low. For example, in Tanzania, the availability of metformin 500 mg was reported to be 100% while in our study it was 64.9%, yet gliclazide 80 mg had 0% availabity in the surveyed facilities in Tanzania compared with 50.8% availability in our study [4]. A study reported that the availability of carbamazepine 200 mg tablets in Ethiopia was 82.8%, whereas it was 43.1% in our study [8]. In Kenya, the availability of amlodipine 5 mg was 31.6%, bisoprolol 5 mg 0.7%, furosemide 40 mg 71.7%, and glibenclamide 5 mg 66.1%, compared to 70.5%, 62.1%, 70.3%, and 43.7%, respectively, in our study [37]. These data suggest that while certain medicines may achieve high availability in some low-income contexts, others remain consistently of low availabity, reflecting systemic constraints such as weak supply chains and limited regulatory capacity.”* (Lines: 507-519 in the manuscript with track changes)

**Table 1- need to explain what the different shades of purple mean**

We agree that the previous use of purple shading could be unclear to readers. To address this, we replaced the purple color gradient with a standardized color-coding scheme and added a clear legend below Table 1 as following:

“*Availability was described using the range: Very low: <30% (red); Low: 30–49% (orange); and Somewhat high: 50–80% (green)*.”

**Figure 1- also need add a figure caption and o explain or label different colored regions**

We would like to clarify that the caption and explanation for Figure 1 are already included in the manuscript text, as required by the PLOS Global Public Health submission guidelines, rather than in the uploaded figure file. The caption reads as follows:

“*Figure 1: Map of Syria, presenting territorial control in Syria as of 2024. Areas in pink represent regions controlled by the Assad government, yellow areas are under the control of the Autonomous Administration of North and East Syria, and green areas are controlled by opposition factions and Turkish-backed groups*.” (Lines: 110-114 in the manuscript with track changes)

According to the journal’s instructions (“Do not include captions as part of the figure files or submit them in a separate document”; https://journals.plos.org/globalpublichealth/s/figures" \l "loc-captions

**Reviewer #3: Introduction:

Line 111 – The term ‘insulin-dependent diabetic’ is obsolete and not used now. Please rephrase it to Type 1 Diabetes patients. The term ‘diabetic’ should also not be used.**
Thank you for this important observation. We have updated the terminology to use clearer and more appropriate phrasing. The term “*insulin-dependent diabetic*” has therefore been revised. We replaced it with “*patients requiring insulin therapy*” (Lines: 106-107 in the manuscript with track changes), as insulin use in may include both Type 1 and insulin-treated Type 2 diabetes cases. We have also replaced other instances of the term “*diabetic*” with “*patients with diabetes*” to ensure consistency and appropriate phrasing.

**The introduction is very long. Suggest to shorten the text by merging paragraphs 3, 4 and 5 with a focus on the problem statements. Lengthy explanations about the country are unnecessary.**

Thank you for this suggestion. We have shortened the introduction by merging paragraphs 3–5 into one paragraph focused on the health system collapse, territorial fragmentation, and access to medicines. Please see lines: 82-109 in the manuscript with track changes

Discussion:

**555-559 – There is mention of a manufacturer’s name, “Novo Nordisk’. This name can be avoided as this is a scientific manuscript. Explaining the issue would suffice, or use a more general term eg, ‘the insulin supplier’.**
Thank you for this observation. We have removed the reference to “Novo Nordisk” and as suggested, we replaced it with a more general description, referring instead to “*insulin manufacturers*” (Lines: 568-569 in the manuscript with track changes)

**609 – ‘At the moment of writing this paper’ is inappropriate as this is a scientific manuscript. Suggest rephrasing using other terms, eg, ‘Currently’ etc.**

Thank you for pointing this out. We have replaced this with “currently”(Line: 521 in the manuscript with track changes)
